# Morphological measurements in computed tomography correlate with airflow obstruction in chronic obstructive pulmonary disease: systematic review and meta-analysis

**DOI:** 10.1007/s00330-012-2480-8

**Published:** 2012-06-15

**Authors:** XueQian Xie, Pim A. de Jong, Matthijs Oudkerk, Ying Wang, Nick H. T. ten Hacken, Jingtao Miao, GuiXiang Zhang, Geertruida H. de Bock, Rozemarijn Vliegenthart

**Affiliations:** 1 Center for Medical Imaging–North East Netherlands (CMI-NEN), Department of Radiology, University of Groningen, University Medical Center Groningen, Hanzeplein 1, P.O. Box 30.001, 9700 RB Groningen, The Netherlands; 2 Department of Radiology, University Medical Center Utrecht, University of Utrecht, Heidelberglaan 100, P.O. Box 85.500, 3584 CX Utrecht, The Netherlands; 3 Department of Radiology, Tianjin Medical University General Hospital, Anshan Road 154, 300052 Tianjin, China; 4 Department of Pulmonary Diseases, University of Groningen, University Medical Center Groningen, Hanzeplein 1, P.O. Box 30.001, 9700 RB Groningen, The Netherlands; 5 Department of Radiology, Shanghai Jiao Tong University Affiliated First People’s Hospital, Haining Road 100, 200080 Shanghai, China; 6 Department of Epidemiology, University of Groningen, University Medical Center Groningen, Hanzeplein 1, P.O. Box 30.001, 9700 RB Groningen, The Netherlands

**Keywords:** Review, systematic, Meta-analysis, Tomography, X-ray computed, Pulmonary disease, chronic obstructive, Function test, pulmonary

## Abstract

**Objectives:**

To determine the correlation between CT measurements of emphysema or peripheral airways and airflow obstruction in chronic obstructive pulmonary disease (COPD).

**Methods:**

PubMed, Embase and Web of Knowledge were searched from 1976 to 2011. Two reviewers independently screened 1,763 citations to identify articles that correlated CT measurements to airflow obstruction parameters of the pulmonary function test in COPD patients, rated study quality and extracted information. Three CT measurements were accessed: lung attenuation area percentage < -950 Hounsfield units, mean lung density and airway wall area percentage. Two airflow obstruction parameters were accessed: forced expiratory volume in the first second as percentage from predicted (FEV_1_ %pred) and FEV_1_ divided by the forced volume vital capacity.

**Results:**

Seventy-nine articles (9,559 participants) were included in the systematic review, demonstrating different methodologies, measurements and CT airflow obstruction correlations. There were 15 high-quality articles (2,095 participants) in the meta-analysis. The absolute pooled correlation coefficients ranged from 0.48 (95 % CI, 0.40 to 0.54) to 0.65 (0.58 to 0.71) for inspiratory CT and 0.64 (0.53 to 0.72) to 0.73 (0.63 to 0.80) for expiratory CT.

**Conclusions:**

CT measurements of emphysema or peripheral airways are significantly related to airflow obstruction in COPD patients. CT provides a morphological method to investigate airway obstruction in COPD.

**Key Points:**

*• Computed tomography is widely performed in patients with chronic obstructive pulmonary disease (COPD)*

*• CT provides quantitative morphological methods to investigate airflow obstruction in COPD*

*• CT measurements correlate significantly with the degree of airflow obstruction in COPD*

*• Expiratory CT measurements correlate more strongly with airflow obstruction than inspiratory CT*

*• Low-dose CT decreases the radiation dose for diagnosis and quantitative emphysema evaluation*

**Electronic supplementary material:**

The online version of this article (doi:10.1007/s00330-012-2480-8) contains supplementary material, which is available to authorized users.

## Introduction

Chronic obstructive pulmonary disease (COPD) is characterised by airflow limitation that is not fully reversible [1]. The pathogenesis of airflow limitation in COPD is mainly related to emphysema and small airway remodelling [2]. Although airflow obstruction parameters in the pulmonary function test (PFT) by spirometry is essential in COPD diagnosis, these parameters fail to quantify the proportionate impact of emphysema and small airways disease individually. Morphological changes can be characterised and quantified by computed tomography (CT), especially by multi-detector CT [3]. For COPD patients, quantitative chest CTs are important for understanding the pathogenesis and the effect of therapeutic interventions [4], and can help to identify those most at risk for acute exacerbations [5]. Since the introduction of the ‘density mask’ in 1988, CT emphysema quantification has had a long history [6–8]. Multi-detector CT can accurately evaluate emphysema [9]. However, quantification of airway remodelling by CT is challenging because of its spatial resolution. Airway wall quantification started over a decade ago, mainly for large airways [3], but investigators have measured the peripheral airways down to 0.5-mm-lumen diameter [10] and 2.8 mm outer diameter [11]. Measurement of narrowing of CT-detectable airways may estimate the degree of small airways disease [10].

The assumption is that emphysema and peripheral airway wall thickness, as detected by CT, are correlated to airflow obstruction in COPD patients. Study results have been variable and sometimes conflicting. However, some individual studies have been small and underpowered [2, 12–15]. Therefore, we conducted a systemic review and meta-analysis to determine the correlation between emphysema or peripheral airway measurements on inspiratory and expiratory CT, and airflow obstruction in COPD.

## Materials and methods

This study was conducted according to Preferred Reporting Items for Systematic Reviews and Meta-analyses (PRISMA) [16].

### Data sources and searches

We searched PubMed, Embase and Web of Knowledge from January 1976 to December 2011, from the start of whole-body CT, using terms related to computed tomography and PFT (i.e. lung function*, respiratory function*, pulmonary function*, etc.) and COPD (i.e. chronic obstructive pulmonary disease*, chronic obstructive lung disease*, etc.) without language restrictions (Electronic supplementary Table 1). Unpublished studies were not included.

### Study selection

Four reviewers with at least 6 years' experience in thoracic radiology participated in the study selection. Each study was evaluated independently by two reviewers out of three, with disagreements resolved by the fourth reviewer. Articles were included in the systematic review if they: (1) analysed the association between CT quantitative emphysema or airway measurements and PFT; (2) investigated human beings; (3) included participants diagnosed with stable adult COPD, according to the Global initiative for chronic Obstructive Lung Disease (GOLD) [1] or the American Thoracic Society (ATS) or the European Respiratory Society (ERS), or clearly defined similar criteria; (4) included participants who had clearly described PFT, according to the guidelines of the ATS, ERS or similar methods. Articles were excluded if they: (1) were reviews, abstracts, case reports or letters; (2) were laboratory or phantom studies; (3) covered participants with confounding disease, such as interstitial lung disease, chronic bronchitis, asthma and α-1 anti-trypsin disease.

Articles were subsequently included in the meta-analysis if they: (1) had no selection bias (e.g. only mild or only severe COPD); (2) had a sample size of ≥20 (20 subjects would provide a power of 0.90 when detecting a typical effect correlation coefficient of 0.60); (3) were performed using volumetric multidetector CT; (4) reported correlation coefficients; (5) reported the percentage of lung attenuation area under -950 HU (%LAA-950), mean lung density (MLD) or wall area percentage (WA%) in airways ≥ fifth airway generation (sub-sub-segment level) as CT measurements; (6) reported the predicted forced expiratory volume in the first second as percentage (FEV_1_ %pred) and FEV_1_ divided by the forced volume vital capacity (FEV_1_/FVC) as spirometry parameters. Studies were excluded if the CT examination only included selected pulmonary levels or if the slice increment was larger than the slice thickness. In possible duplicate reports, the report with the largest sample size was included.

### Data extraction and quality assessment

Two reviewers evaluated independently, with disagreements resolved by a third reviewer. A standardised extraction form was used to collect study characteristics, participant characteristics, methodology and correlation coefficients. The systematic review included ten CT measurements: %LAA-960, %LAA-950, %LAA-910, %LAA-900, MLD, 15 percentile point of lung density (Perc15), lung volume (LV), WA%, airway wall thickness (WT) and airway lumen area (Ai). Three CT measurements, %LAA-950, MLD and WA% were pooled in the meta-analysis. Two PFT parameters for airflow obstruction were collected, including FEV_1_ %pred and FEV_1_/FVC.

Methodological quality and potential sources of bias of the included meta-analysis articles were assessed with 14 standard items of the Quality Assessment of Diagnostic Accuracy Studies (QUADAS) tool [17]. For each article, a quality score was accumulated by assigning 1 point to each fulfilled QUADAS item, 0.5 to unclear items and 0 to unmet items. A score of ≥ 11 points was considered high quality and a score < 11 points as low quality. Cohen’s *k* was calculated to indicate inter-observer agreement. Publication bias was evaluated with Begg and Mazumdar rank correlation, Egger’s regression test and Rosenthal fail-safe n test.

### Data synthesis and analysis

Summary measure was the correlation coefficient (CC). Pooled CCs with 95 % confidence intervals (CIs) were calculated using Hedges-Vevea random effects model and Z-test for normality. Pooled CCs were calculated for the correlations between %LAA-950 and FEV_1_ %pred, %LAA-950 and FEV_1_/FVC, MLD and FEV_1_ %pred, MLD and FEV_1_/FVC, and WA% and FEV_1_ %pred in inspiratory and expiratory CT. If multi-level bronchi were evaluated, we chose the smallest bronchi. Heterogeneity was tested using the Q statistic and I^2^ index. The random effects model was used regardless of the heterogeneity test, although results in the Q statistic were still stated. To investigate the impact of individual variables on the meta-analysis results, subgroup analysis was performed if a subgroup consisted of at least two studies. Subgroups were based on radiation dose (low or normal dose) and breath-hold procedure (inspiratory or expiratory). Meta-regression was performed to investigate the influence of gender, if the male percentage was reported by at least three studies. Statistical analysis was performed using SPSS 18.0 (IBM, NY, USA) and R 2.12.0 (R Foundation, Vienna, Austria). *P* < 0.05 was considered statistically significant.

## Results

### Study selection

**Fig. 1:**
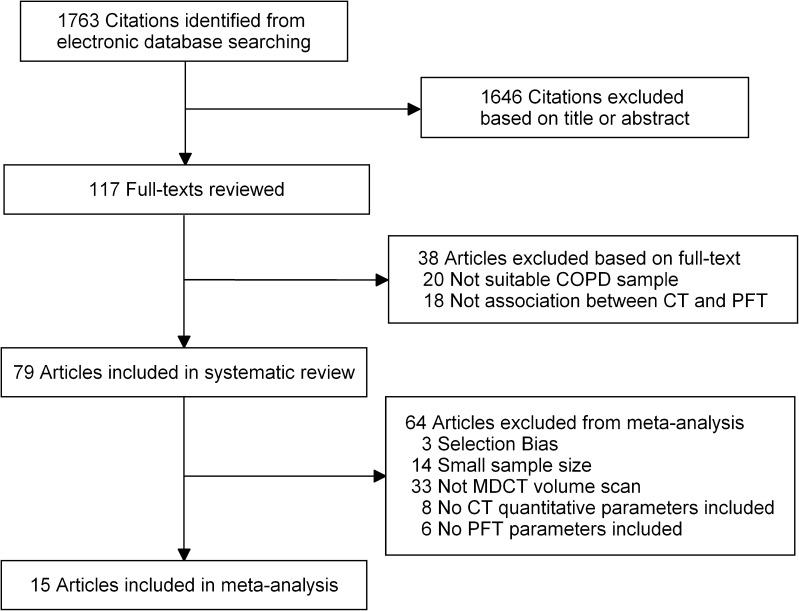
Flowchart of literature review and selection. COPD = Chronic obstruction pulmonary disease; PFT = pulmonary function test; MDCT = multi-detector computed tomography

The database searches elicited 1,763 citations (Fig. 1). Seventy-nine articles were included in the systematic review and 15 articles [13, 18–31] in the meta-analysis, including 10 [18–23, 25, 26, 28, 29] from trial cohorts.

### Systematic review

The systematic review included 9,559 COPD participants (range of mean age, 48 to 73 years), including 6,101 (63.8 %) men, 2,000 (20.9 %) women and 1,458 (15.3 %) without indicated gender (Electronic supplementary Table 2). A total of 6,935 (72.5 %) were (ex-)smokers and 133 (1.4 %) non-smokers; for 2,491 (26.1 %) no smoking status was reported. Fifty-four (68.4 %) studies were prospective and 23 (29.1 %) retrospective; in 2 (2.5 %) articles the study design was not reported. Forty-one (51.9 %) articles were from Europe, 23 (29.1 %) from Asia and 15 (19.0 %) from North America. Of the articles, 69 (87.3 %) were written in English, 5 (6.3 %) in Italian, 3 (3.8 %) in Chinese, 1 (1.3 %) in French and 1 (1.3 %) in Polish.

**Fig. 2:**
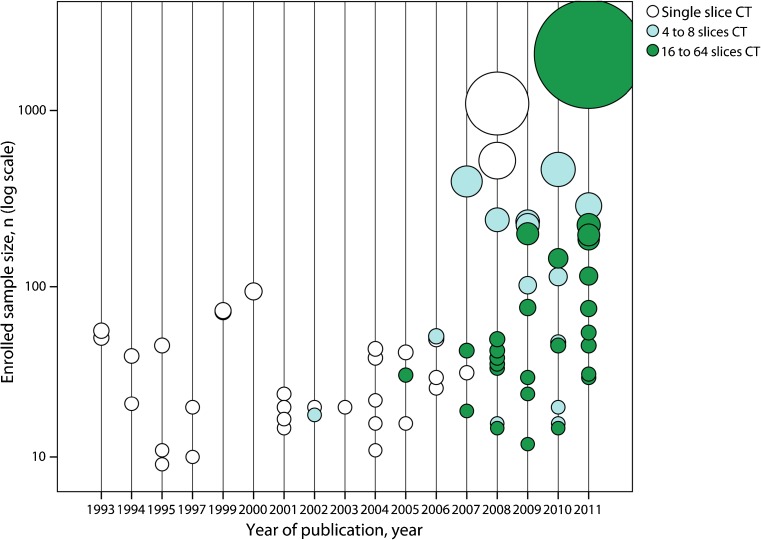
Sample size of the articles included in the systematic review by year of publication and CT generation. MDCT = Multi-detector computed tomography

The tendency was towards larger sample size and more advanced CT equipment in recent publications (Fig. 2). Before 2007, only articles with sample size <100 were found. After 2007, larger sample size articles were published. Although single-slice CT (32 articles, 40.5 %) was continuously used from 1993 to 2009, multi-detector CT has been popular in recent years. Since 2002, 4- and 8-slice multi-detector CT (16 articles, 20.2 %) has been used. Since 2005, 16- and 64-slice multi-detector CT (29 articles, 36.7 %) has been used.

Included articles varied in methodology. Volume acquisition was used in 36 articles (45.6 %) and non-volume acquisition in 43 (54.4 %). Selected slices were acquired in 21 studies (26.6 %) and whole lung in 58 (73.4 %). Median slice thickness was 1.0 mm (range, 0.625-10 mm). Median slice increment was 2.0 mm (range, 0.625-20 mm). Low radiation dose was used in 12 articles (15.2 %) and normal dose in 67 (84.7 %). Both inspiratory and expiratory CT findings were evaluated in 24 articles (30.4 %), and only inspiratory in 54 articles (68.3 %) and expiratory in 1 article (1.3 %).

Sixty-four different CT measurements and 27 different PFT parameters were reported (Electronic supplementary Figs. 4 and 5). Common CT measurements were %LAA-950 in 36 (45.6 %) studies, MLD in 22 (27.8 %), WA% in 17 (21.5 %) and visual score in 17 (21.5 %). Common PFT parameters were FEV_1_ %pred in 72 articles (91.1 %) and FEV_1_/FVC in 64 articles (81.0 %). Common lung parenchyma thresholds defining emphysema ranged from -900 HU to -960 HU, with the most commonly used threshold being -950 HU. In some studies, different correlations to airflow obstruction parameters in PFT were found with these differing thresholds in the same sample [12, 14, 27, 32]. The CC between %LAA-950 and FEV_1_ %pred ranged from -0.67 to -0.09 [12, 13], between MLD and FEV_1_ %pred from 0.18 to 0.85 [12, 33], between WA% and FEV_1_ %pred from -0.713 to -0.044 [22, 34], between %LAA-950 and FEV_1_/FVC -0.75 to -0.09 [12, 35], and between MLD and FEV_1_/FVC from 0.21 to 0.89 [12, 33]. In four articles, the CC between Perc15 and FEV_1_ %pred in inspiration ranged from 0.09 to 0.62, and the CC between Perc15 and FEV_1_/FVC in inspiration from 0.12 to 0.62 [12, 13, 36, 37] (Electronic supplementary Table 3).

### Risk of bias in the meta-analysis

All articles included in the meta-analysis were high quality (Electronic supplementary Table 4 and Electronic supplementary Fig. 6). The quality score ranged from 12.5 to 13.5. Suboptimal scores were present for three QUADAS items: 7 articles without an interval between CT and PFT (item 4), 9 articles without an indication whether CT quantification was blinded to PFT (item 10) and 13 articles without an indication whether PFT was blinded to CT quantification (item 11). Cohen’s *k* was 0.925, expressing very good inter-observer agreement. No publication bias was found (Electronic supplementary Table 5). The median of Rosenthal fail-safe n was 122 (range, 84 to 614), indicating a solid empirical result.

Three of the nine meta-analysis calculations showed mild heterogeneity. The I^2^ index was > 50 % for the correlation between MLD and FEV_1_ %pred in inspiration (*P* = 0.11, I^2^ index = 50.6 %), between MLD and FEV_1_/FVC in inspiration (*P* = 0.02, I^2^ index = 75.9 %), and between WA% and FEV_1_ %pred (*P* = 0.04, I^2^ index = 64.7 %).

### Synthesis of results in the meta-analysis

**Fig. 3:**
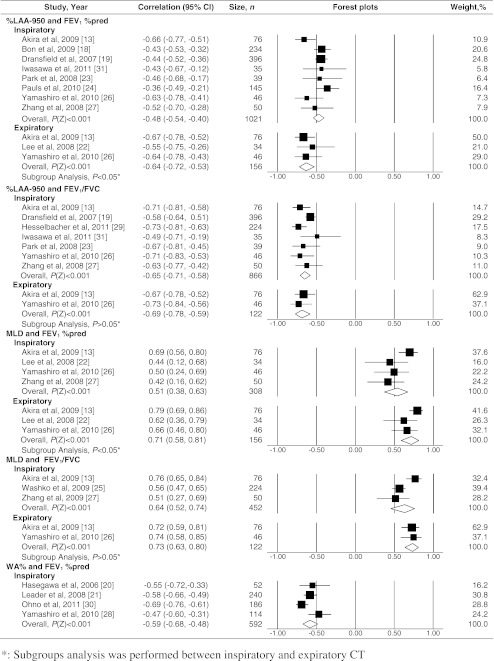
Forest plots for correlations between CT measurements and airflow obstruction. CI = Confidence interval; *P*(Z) = *P* value of Z test; FEV_1_ %pred = percentage of the predicted forced expiratory volume in the first second; FEV_1_/FVC = FEV_1_ divided by forced vital capacity; %LAA-950 = percentage lower attenuation area than -950 HU; MLD = mean lung density; Perc15 = 15 percentile point of lung density; WA% = wall area percentage

The meta-analysis included 2,095 participants out of 9,559 in the systematic review (Fig.3).

Nine articles [13, 18, 19, 22–24, 26–28, 31] reported CC between %LAA-950 and FEV_1_ %pred in inspiration. Two [19, 25] were from the National Lung Screening Trial (NLST) cohort and two [22, 23] from the Korean Obstructive Lung Disease (KOLD) cohort. Because of possible duplicate reporting, articles [22, 25] with smaller sample sizes were excluded from each cohort. The pooled CC between %LAA-950 and FEV_1_ %pred was -0.48 (95% CI: -0.54, -0.40) in inspiration and -0.64 (-0.72, -0.53) in expiration. The pooled CC between %LAA-950 and FEV_1_/FVC was -0.65 (-0.71, -0.58) in inspiration and -0.69 (-0.78, -0.59) in expiration.

No potential duplicate report was found for MLD. The pooled CC between MLD and FEV_1_ %pred was 0.51 (95% CI: 0.38, 0.63) in inspiration and 0.71 (0.58, 0.81) in expiration. The pooled CC between MLD and FEV_1_/FVC was 0.64 (0.52, 0.74) in inspiration and 0.73 (0.63, 0.80) in expiration.

Seven articles [18, 20–22, 25, 28, 30] reported CC between WA% and FEV_1_ %pred in inspiration. Two articles [18, 22] were excluded because airway measurements concerned only airways above the fifth airway generation. Another article [25] was excluded because it did not report which airways were measured. Four articles [20, 21, 28, 30] were finally included. The lumen diameter of peripheral airways was about 2-3 mm in the included articles. The pooled CC between WA% and FEV_1_ %pred was -0.59 (95% CI: -0.68, -0.48) in inspiration. Expiratory CT was not used for airway measurements.

### Subgroup analysis and meta-regression

Subgroup analysis for radiation dose was performed for the association between %LAA-950 and FEV_1_ %pred in inspiration, indicating no significant difference (*P* > 0.05). In low dose [18, 19], the pooled CC was -0.44 (95% CI: -0.50, -0.37). In normal dose [13, 23, 24, 26, 27, 31], the pooled CC was -0.50 (-0.57, -0.42). Subgroup analysis was also performed for inspiratory and expiratory CT. A significantly stronger negative correlation was found between %LAA-950 and FEV_1_ %pred in expiratory CT (*P* < 0.05), and a stronger positive correlation between MLD and FEV_1_ %pred (*P* < 0.001), but no difference was found in the association between %LAA-950 and FEV_1_/FVC (*P* > 0.05), or MLD and FEV_1_/FVC (*P* > 0.05). In meta-regression for gender contribution, no statistically significant effect modification was found for male percentage (*P* > 0.05) (Electronic supplementary Table 5).

## Discussion

In this meta-analysis, significant correlations were found between CT measurements of emphysema or peripheral airway and airflow obstruction parameters in PFT in COPD patients, both in inspiratory and expiratory CT. The range of absolute correlation coefficients between included CT measurements and airflow obstruction was 0.48 to 0.65 for inspiratory CT and 0.64 to 0.73 for expiratory CT. These results confirm correlations between morphology and function in COPD patients. The confidence in these findings is strong, as results were based on high methodological quality studies without publication bias. Thus, CT provides a quantitative morphological method to investigate the principle components of airway obstruction in COPD, with similar strength of associations with airflow obstruction for CT measurements of emphysema and peripheral airways. The strongest association was found between CT emphysema measurements and FEV_1_/FVC, especially in expiratory CT. Our systematic review demonstrated differing methodologies for CT quantification and contrasting correlations with airflow obstruction in COPD patients.

CT quantification reflects pathophysiological changes in COPD to some degree. The pathological findings of airway limitation are in airways < 2 mm in internal diameter [38]. Such small airways can hardly be measured directly by CT because of the spatial resolution limit. However, peripheral airway (≥ 5^th^ generation) wall thickness can be measured as WA%. Destruction of the lung parenchyma (emphysema) can be measured as %LAA-950 or MLD. The morphological contribution from these two pathological processes is difficult to distinguish by spirometry, but is important for COPD research. The morphological information in CT quantification of the relative predominance of peripheral airway wall disease or emphysema may in the future allow more focused treatment of the predominating COPD phenotype.

This systematic review incorporated ten different CT measurements. Although a visual score was common in earlier publications, we did not discuss it, because of its subjective nature. Perc15 seems an effective measurement for emphysema, but Perc15 results could not be pooled because of an insufficient study number. Only three (%LAA-950, MLD and WA%) of the ten measurements were eventually pooled because the study number was sufficient to perform a meta-analysis. We investigated two PFT parameters (FEV_1_ %pred and FEV_1_/FVC) as they are the two most commonly used functional parameters regarding airway limitation.

In subgroup analysis, associations for inspiratory and expiratory CT findings were compared. Some authors have reported that CT measurements in expiration are more closely correlated with airflow obstruction than in inspiration [14, 39–41]. Our results indicate that CT measurements in expiration rather than in inspiration were more correlated with FEV_1_ %pred, not with FEV_1_/FVC. Whether an additional expiratory CT data acquisition should be performed for COPD evaluation is debatable. Expiratory CT exposes patients to additional radiation; however, with developments in CT technique, the additional dose will likely decrease. Also, we found low radiation doses did not change correlations between CT emphysema quantification and airflow obstruction compared to normal doses. Low-dose CT can decrease the overall radiation dose for CT quantitative emphysema evaluation without loss in diagnostic value.

Multiple airway generations were included in the systematic review, but only peripheral airways (≥ 5^th^ generation) in the meta-analysis. Some authors investigated airways from the third to fifth or sixth generation, and found that the association between airway wall measurements and PFT was stronger for higher generations than lower generations [20, 28]. Therefore, we only pooled results for airways ≥ fifth generation. Some authors reported moderate associations with larger airways, ranging from -0.39 to -0.54 [18, 42]. In our meta-analysis, the association between wall area percentage of peripheral airways and FEV_1_ %pred was -0.59. One factor to keep in mind is the overestimation of airway wall thickness, showing a relative increase with each airway generation [3]. Despite this factor, based on our pooled results, the association between disease of the more peripheral airways (≥ 5th generation) and lung function appears stronger than for lower generation airways (< 5th generation), suggesting that airway wall thickness measurements on CT should be performed on the smallest airways visible.

This study confirms significant correlations between CT measurements and airflow obstruction in COPD. The correlations were in agreement with some expert narrative reviews [9, 10] and individual studies [14, 15, 39, 42, 43]. Nevertheless, other studies reported weaker associations, e.g. the National Emphysema Treatment Trial (NETT) study [12] and the International COPD Genetics Network (ICGN) study [2]. In NETT and ICGN, predominantly single-slice CT was used. Since single-slice CT decreases reproducibility and accuracy [44], this has likely caused the reduced strength of the correlations.

This study has some limitations. First, no prospective large cohort with up-to-date CT technology was found as primary study. The largest study [45] included over 2,000 participants, but most of the participants had normal lung function. In inspiratory CT, the number of included articles was relatively small, but sufficient to perform a reliable meta-analysis. However, in expiratory CT, some indicators of bias could not be determined because of the limited number of studies. Second, we found 64 different quantitative CT measurements in the literature. We chose ten for systematic review and three for meta-analysis. Although the included parameters are representative, the other 54 measurements could be valuable to evaluate COPD. In another way, FEV_1_ %pred and FEV_1_/FVC were selected as airflow obstruction parameters in PFT, but other parameters in PFT were valuable for evaluating COPD. Third, mild heterogeneity was found in three correlations in the meta-analysis. A random effects model was used to compensate for the heterogeneity.

In conclusion, measurements of emphysema and the peripheral airways on inspiratory and expiratory CT have significant correlations with airflow obstruction as accessed by FEV_1_ %pred and FEV_1_/FVC in COPD patients. Thus, CT provides a quantitative morphological method to investigate airflow obstruction by emphysema and peripheral airway disease in COPD.

## Electronic supplementary material

Below is the link to the electronic supplementary material.

ESM 1 (DOC 78.5 kb)

ESM 2 (DOC 491 kb)

ESM 3 (DOC 434 kb)

ESM 4 (DOC 179 kb)

ESM 5 (DOC 100 kb)

ESM 6 (DOC 77.5 kb)

ESM 7 (DOC 76.5 kb)

ESM 8 (JPEG 36 kb)

ESM 9 (DOC 36.5 kb)

## Abbreviations

%LAA-950: Percentage of lung attenuation area under -950 HU
Ai: Airway lumen area
ATS: American Thoracic Society
CC: Correlation coefficient
CI: Confidence interval
COPD: Chronic obstructive pulmonary disease
CT: Computed tomography
ERS: European Respiratory Society
FEV_1_ %pred: Forced expiratory volume in the first second as percentage from predicted
FEV_1_/FVC: FEV_1_ divided by the forced volume vital capacity
GOLD: Global initiative for chronic Obstructive Lung Disease
HU: Hounsfield unit
LV: Lung volume
MLD: Mean lung density
Perc15: 15 percentile point of lung density
PFT: Pulmonary function test
PRISMA: Preferred Reporting Items for Systematic Reviews and Meta-analyses
QUADAS: Quality Assessment of Diagnostic Accuracy Studies
WA%: Wall area percentage
WT: Airway wall thickness
