# Supplementary material for: Morphological measurements in computed tomography correlate with airflow obstruction in chronic obstructive pulmonary disease: systematic review and meta-analysis
Source: Eur Radiol. 2012 Jun 15;22(10):2085–93. doi: 10.1007/s00330-012-2480-8 (PMC3431473; doi:10.1007/s00330-012-2480-8)
Supplement: Supplementary file 1 — (DOC 78.5 kb) [file 330_2012_2480_MOESM1_ESM.doc]

**Electronic supplementary table 1 Literature search strategy. Last search on January 5, 2012**

| **Search terms used to identify relevant citations**  A: Computed tomography  CT  B: Lung function*  Respiratory function*  Pulmonary function*  Spirometr*  Diffusing capacity  Diffusion capacity  Airway obstruction parameter*  C: Chronic obstructive pulmonary disease*  COPD  Chronic obstructive lung disease*  Chronic airflow obstruction*  To search: A and B and C  The term ‘CT’ is restrained to the title and abstract  Publication date: From January 1976 to December 2011  **Search Query for PubMed**  #1: ("Tomography, X-Ray Computed"[MeSH] OR "computed tomography"[TIAB] OR CT[TIAB])  #2: ("Pulmonary Disease, Chronic Obstructive"[MeSH] OR COPD OR "chronic obstructive pulmonary disease*" OR "chronic obstructive lung disease*" OR "Chronic airflow obstruction*")  #3: ("Pulmonary Function Test" OR Spirometr* OR "pulmonary function*" OR "Lung function*" OR "Respiratory function*" OR "Diffusing capacity" OR "Diffusion capacity" OR "Airway obstruction parameter*")  Grammar in advanced search: 1976/01:2011/12 [dp] and #1 and #2 and #3  **Search Query for Embase**  #1: ((Computed tomography):ab,ti OR CT:ab,ti)  #2: ((Chronic Obstructive Pulmonary Disease*) OR COPD OR (Chronic Obstructive Lung Disease*) OR (Chronic obstructive airway disease*) OR (Chronic airflow obstruction*))  #3: ('lung function test'/exp OR (Spirometr*) OR ('Lung function') OR ('Pulmonary function') OR ('Respiratory function') OR ('Lung functions') OR ('Pulmonary functions') OR ('Respiratory functions') OR ('Diffusing capacity') OR ('Diffusion capacity') OR ('Airway obstruction'))  Grammar in advanced search: #1 AND #2 AND #3 AND [1-1-1976]/sd NOT (#1 AND #2 AND #3 AND [31-12-2011]/sd)  **Search Query for Web of Knowledge**  #1 topic: ((Computed tomography) OR CT)  #2 topic: ((Chronic Obstructive Pulmonary Disease*) OR COPD OR (Chronic Obstructive Lung Disease*) OR (Chronic obstructive airway disease*) OR (Chronic airflow obstruction*))  #3 topic: (Spirometr* OR (Lung function*) OR (Pulmonary function*) OR (Respiratory function*) OR (Diffusing capacity) OR (Diffusion capacity) OR (Airway obstruction parameter*))  Grammar: #1 topic and #2 topic and #3 topic |
| --- |
