# Supplementary material for: Morphological measurements in computed tomography correlate with airflow obstruction in chronic obstructive pulmonary disease: systematic review and meta-analysis
Source: Eur Radiol. 2012 Jun 15;22(10):2085–93. doi: 10.1007/s00330-012-2480-8 (PMC3431473; doi:10.1007/s00330-012-2480-8)
Supplement: Supplementary file 2 — (DOC 491 kb) [file 330_2012_2480_MOESM2_ESM.doc]

**Electronic supplementary table 2 Characteristics of studies included in the systematic review**

| **Study, Year** | **Patients, *n*** | **Men, %** | **Age,**  **Year ± SD or**  **year (range)** | **COPD Severity,**  **GOLD stage or**  **FEV_1_ %pred ± SD or FEV_1_ %pred (range)** | **CT type, slice** | **Inspiratory or expiratory CT examination** | **Volumetric CT examination** | **Radiation Dose** | **Cohort Name** |
| --- | --- | --- | --- | --- | --- | --- | --- | --- | --- |
| Achenbach et al, 2008 [42] | 16 | 75 | 65(50-75) | Stage II-III | 4 | Inspiratory | Volume | Normal |  |
| Akira et al, 2009 [13]* | 76 | 88 | 67(37-85) | Stage 0-IV | 16 | Both | Volume | Normal |  |
| Bae et al, 1997 [S1] | 10 | NA | 57(41-77) | NA | 1 | Both | Non-volume | Normal |  |
| Bafadhel et al, 2011 [S2] | 75 | 77 | 67(43-88) | 47±2%^†^ | 16 | Inspiratory | Non-volume | Normal | BCOPDE |
| Baldi et al, 2001 [S3] | 24 | 75 | 61±11 | 35%(17-72%) | 1 | Inspiratory | Non-volume | Normal |  |
| Beinert et al, 1995 [S4] | 11 | 82 | 48(34-56) | 41±0.04%^†^ | 1 | Both | Non-volume | Normal |  |
| Bon et al, 2009 [18]* | 234 | 50 | 61(50-78) | Stage 0-IV | 4&8 | Inspiratory | Volume | Low | PLuSS |
| Boschetto et al, 2006 [43] | 26 | 85 | 71±3(*n*=11)^†^  70±2(*n*=15)^†^ | 31±2.6%(*n*=11)^†^  47±3.8%(*n*=15)^†^ | 1 | Inspiratory | Non-volume | Normal |  |
| Camiciottoli et al, 2006 [39] | 51 | 90 | 64(43-78) | 52%(15-106%) | 1 | Both | Non-volume | Normal |  |
| Cavigli et al, 2009 [S5] | 30 | 77 | 68(52-81) | Stage I-IV | 16 | Inspiratory | Volume | Normal |  |
| Cerveri et al, 2004 [S6] | 39 | 90 | 64(48-80) | 47%(21-72%) | 1 | Expiratory | Non-volume | Normal |  |
| Crausman et al, 1995 [S7] | 9 | 22 | 67±2^†^ | 40%(20-89%) | 1 | Inspiratory | Non-volume | Normal |  |
| Daghfous et al, 1993 [S8] | 51 | 90 | 55(25-80) | 40±21%(*n*=31)  39±17%(*n*=20) | 1 | Inspiratory | Non-volume | NA |  |
| D’Anna et al, 2011 [S9] | 59 | 68 | 68±7 | 52±18 | 16 | Inspiratory | Non-volume | Normal |  |
| Demir et al, 2005 [S10] | 16 | 100 | 65±6 | 41±15% | 1 | Inspiratory | Non-volume | NA |  |
| Deveci et al, 2004 [34] | 22 | 100 | 57±5(*n*=17)  61±7(*n*=5) | Stage II-III | 1 | Inspiratory | Non-volume | Normal |  |
| Dransfield et al, 2007 [19]* | 396 | 62 | 63±5(*n*=246)  61±5(*n*=150) | Stage 0-IV | Multi | Inspiratory | Volume | Low | NLST |
| Falaschi et al, 1995 [33] | 46 | 83 | 63(46-78) | NA | 1 | Both | Non-volume | Normal |  |
| Gelb et al, 1993 [S11] | 56 | 48 | 68(53-76) | 46%(18-79%) | 1 | Inspiratory | Non-volume | Normal |  |
| Grydeland et al, 2010 [S12] | 463 | 65 | 65±9(*n*=299)  63±9(*n*=164) | Stage II-IV | 8 | Inspiratory | Non-volume | Normal | GenKOLS |
| Grydeland et al, 2011 [S13] | 288 | 70 | 64±10(*n*=202)  60±8(*n*=86) | Stage II-IV | 8 | Inspiratory | Non-volume | Normal | GenKOLS |
| Hasegawa et al, 2006 [20]* | 52 | 96 | 72(41-84) | Stage I-IV | 4 | Inspiratory | Volume | Normal | Hokkaido |
| Hesselbacher et al, 2011 [29]* | 224 | 65 | >40 | Stage I-IV | 64 | Inspiratory | Volume | Normal | LESCOPD |
| Heussel et al, 2009 [36] | 102 | NA | 64(20-87) | Stage III-IV | 4 | Inspiratory | Volume | Normal |  |
| Iwasawa et al, 2007 [35] | 19 | 100 | 71±7 | Stage II-IV | 16 | Inspiratory | Volume | Normal |  |
| Iwasawa et al, 2011 [31]* | 35 | 100 | 70±6 | Stage I-IV | 16 | Inspiratory | Volume | Normal |  |
| Jin et al, 2007 [32] | 43 | 51 | 65(45-85) | NA | 16 | Both | Volume | Normal |  |
| Jogi et al, 2011 [S14] | 30 | 65 | 65(53-76) | 51%(25-81%) | Multi | Inspiratory | Volume | NA |  |
| Kim et al, 2009 [S15] | 338 | 64 | 68±6 | <45% | 1 | Inspiratory | Non-volume | Normal | NETT |
| Kosciuch et al, 2009 [11] | 12 | 58 | 57±9 | 72±19% | 16 | Inspiratory | Non-volume | Normal |  |
| Lamers et al, 1994 [8] | 40 | 83 | 60±8(*n*=20)  70±7(*n*=20) | 46±12%(*n*=20)  51±14%(*n*=20) | 1 | Both | Non-volume | Normal |  |
| Leader et al, 2008 [21]* | 240 | NA | NA | Stage 0-IV | 4(*n*=112)  8(*n*=128) | Inspiratory | Volume | Low | PLuSS |
| Leader et al, 2009 [S16] | 200 | NA | NA | Stage 0-IV | 64 | Inspiratory | Volume | Normal | PLuSS |
| Lee et al, 2008 [22]* | 34 | 97 | 65(50-78) | 45%(17-82%) | 16 | Both | Volume | Normal | KOLD |
| Lee et al, 2011 [S17] | 197 | 96 | 67±7(*n*=126)  65±8(*n*=71) | 44±15%(*n*=126)  55±15%(*n*=71) | 16 | Both | Volume | Normal | KOLD |
| Lee et al, 2011 [S18] | 115 | NA | 65 | Stage I-IV | 16 | Both | Volume | Normal | KOLD |
| Li et al, 2009 [S19] | 24 | 75 | 56(32-70) | 56±16% | 64 | Both | Non-volume | Normal |  |
| Madani et al, 2010 [S20] | 16 | 63 | 62(48-83) | Stage I-IV | 4 | Both | Non-volume | Normal |  |
| Marquez-Martin et al, 2011 [S21] | 64 | NA | 64±7 | Stage I-IV | NA | Inspiratory | Non-volume | Normal |  |
| Matsuoda et al, 2007 [14] | 32 | 88 | 73(57-89) | Stage 0-IV | 1 | Both | Non-volume | Normal |  |
| Matsuoda et al, 2008 [S22] | 50 | 80 | 70(57-89) | Stage I-IV | 64 | Both | Volume | Normal |  |
| Matsuoda et al, 2008 [S23] | 36 | 86 | 71(57-89) | Stage I-IV | 64 | Both | Volume | Normal |  |
| Mets et al, 2011 [S24] | 198 | NA | about 60 | Stage I-IV | 16 | Both | Volume | Low | NELSON |
| Mishima et al, 1999 [S25] | 72 | NA | NA | NA | 1 | Inspiratory | Non-volume | Both |  |
| Mohamed Hoesein et al, 2011 [45] | 2085 | 2085 | 60±5 | Stage 0-III | 16 | Inspiratory | Volume | Low | NELSON |
| Moron et al, 2004 [S26] | 16 | 63 | 62±9 | 40±18% | 1 | Inspiratory | Non-volume | NA |  |
| Moroni et al, 2001 [S27] | 20 | 95 | 63(42-73) | NA | 1 | Both | Non-volume | Normal |  |
| Nakano et al, 1999 [S28] | 73 | NA | 69±6 | 46±20% | 1 | Inspiratory | Non-volume | Normal |  |
| Nakano et al, 2000 [3] | 94 | NA | NA | 48%(8-124%) | 1 | Inspiratory | Non-volume | Normal |  |
| O'Donnel et al, 2004 [40] | 44 | NA | 50±7(*n*=17)  57±7(*n*=10)  55±7(*n*=17) | Stage 0-IV | 1 | Both | Non-volume | Normal |  |
| Ohara et al, 2006 [S29] | 30 | 100 | 69±8 | 41±16% | 1 | Inspiratory | Non-volume | Normal |  |
| Ohno et al, 2011 [30]* | 186 | 65 | (23-87) | Stage 0-IV | 16&64 | Inspiratory | Volume | Normal |  |
| Orlandi et al, 2004 [15] | 11 | 82 | 68(60-75) | NA | 1 | Inspiratory | Non-volume | Both |  |
| Orlandi et al, 2005 [S30] | 42 | 88 | 63(42-73) | 49%(15-83%) | 1 | Inspiratory | Non-volume | Normal |  |
| Park et al, 2008 [23]* | 39 | 92 | 66(51-79) | 44±15% | 16 | Inspiratory | Volume | Normal | KOLD |
| Patel et al, 2008 [2] | 519 | 51 | 58±5 | <60% | 1 | Inspiratory | Non-volume | Normal | ICGN |
| Pauls et al, 2010 [24]* | 145 | NA | NA | Stage I-IV | 16 | Inspiratory | Volume | Normal |  |
| Pescarolo et al, 2008 [S31] | 43 | 58 | 62(44-81) | Stage 0-IV | 16&64 | Inspiratory | Volume | Normal |  |
| Petersen et al, 2010 [S32] | 152 | NA | NA | ≥Stage II | 16 | Inspiratory | Volume | Low | DLCST |
| Sandek et al, 2002 [S33] | 20 | 40 | 60±8 | 38±16% | 1 | Both | Non-volume | Normal |  |
| Scichilone et al, 2008 [S34] | 15 | NA | 69(53-90) | Stage I-IV | 40 | Inspiratory | Volume | Normal |  |
| Shaker et al, 2005 [37] | 42 | 38 | 63±8 | 48±13% | 4 | Inspiratory | Volume | Low |  |
| Sorensen et al, 2010 [S35] | 20 | NA | 64(49-80) | 57%(37-76%) | 4 | Inspiratory | Volume | Normal |  |
| Spiropoulos et al, 2003 [S36] | 20 | 90 | 59±9 | 57±26% | 1 | Both | Non-volume | NA |  |
| Torres et al, 2011 [S37] | 115 | 84 | 63±10 | 75±15% | 64 | Inspiratory | Volume | Low |  |
| Tsushima et al, 2010 [S38] | 48 | 83 | 61±9 | Stage I-III | 4 | Inspiratory | Non-volume | Low | Azumi |
| Van Der Lee et al, 2006 [S39] | 50 | 58 | 60(29-83) | 46±24% | 1 | Inspiratory | Non-volume | Normal |  |
| Washko et al, 2008 [12] | 1094 | 61 | 67±6 | <45% | 1 | Inspiratory | Non-volume | Normal | NETT |
| Washko et al, 2009 [25]* | 224 | 42 | 62±5 | Stage I-IV | 4 | Inspiratory | Volume | Low | NLST |
| Watanuki et al, 1994 [S40] | 21 | NA | 65(38-77) | <70% | 1 | Inspiratory | Non-volume | NA |  |
| Yamashiro et al, 2010 [26]* | 46 | 57 | 68(46-81) | Stage 0-IV | 16 | Both | Volume | Normal | LTRC |
| Yamashiro et al, 2010 [28]* | 114 | 57 | 62(56-74) | Stage I-IV | 4 | Inspiratory | Volume | Low | NLST |
| Yamashiro et al, 2011 [S41] | 46 | 57 | 68(46-81) | Stage 0-IV | 16 | Both | Volume | Normal | LTRC |
| Zampatori et al, 1997 [S42] | 20 | 80 | 69(61-86) | 33%(21-57%) | 1 | Both | Non-volume | Normal |  |
| Zampatori et al, 2001 [S43] | 15 | 80 | 63 | 32%(22-63%) | 1 | Inspiratory | Non-volume | Normal |  |
| Zampatori et al, 2001 [S44] | 17 | 77 | 66(47-78) | 47±25% | 1 | Inspiratory | Non-volume | Normal |  |
| Zampatori et al, 2002 [S45] | 18 | 67 | 61(27-81) | <71% | 4 | Inspiratory | Non-volume | Normal |  |
| Zaporozhan et al, 2005 [41] | 31 | 71 | 60(41-76) | Stage II-IV | 16 | Both | Volume | Normal |  |
| Zhang et al, 2008 [27]* | 50 | 66 | 67±10 | Stage 0-IV | 16 | Inspiratory | Volume | Normal |  |

SD = Standard deviation; SE = Standard error; GOLD = The Global Initiative for Chronic Obstructive Lung Disease; FEV_1_ %pred = Predicted forced expiratory volume in the first second; PFT = Pulmonary function test; NA = Not available; VC = Vital capacity; BCOPDE = Biomarkers in COPD exacerbation; PLuSS = the Pittsburgh Lung Screening Study; NLST = the National Lung Screening Trial; GenKOLS = the Genetic COPD Study; LES-COPD = Longitudinal Exacerbation Study of COPD; NETT = the National Emphysema Treatment Trial; KOLD = the Korean Obstructive Lung Disease; NELSON = the Dutch-Belgian Lung Cancer Screening Trial; ICGN = the International COPD Genetics Network; DLCST = the Danish Lung Cancer Screening Trial; LTRC = the National Heart, Lung and Blood Institute Lung Tissue Research Consortium.

* Included in the meta-analysis.

† Expressed as mean ± standard error.
