# Supplementary material for: Morphological measurements in computed tomography correlate with airflow obstruction in chronic obstructive pulmonary disease: systematic review and meta-analysis
Source: Eur Radiol. 2012 Jun 15;22(10):2085–93. doi: 10.1007/s00330-012-2480-8 (PMC3431473; doi:10.1007/s00330-012-2480-8)
Supplement: Supplementary file 3 — (DOC 434 kb) [file 330_2012_2480_MOESM3_ESM.doc]

**Electronic supplementary table 3 Correlation coefficients between CT measurements and airflow obstruction parameters of pulmonary function test in the systematic review**

| **Study, Year** | **CT Measurements** | **Correlation coefficients between CT measurements and airflow obstruction, *r*** | |
| --- | --- | --- | --- |
|  |  | **Inspiratory scan** | **Expiratory scan** |
| Achenbach et al, 2008 [42] | WA%, WA | WA% and FEV_1_ %pred : -0.537  WA and FEV_1_ %pred : -0.423 | NA |
| Akira et al, 2009 [13]* | %LAA-950, -910, MLD, Perc15 Visual score, etc. | %LAA-950 and FEV_1_ %pred: -0.659  %LAA-950 and FEV_1_/FVC: -0.712  %LAA-910 and FEV_1_ %pred: -0.601  %LAA-910 and FEV_1_/FVC: -0.661  MLD and FEV_1_ %pred: 0.694  MLD and FEV_1_/FVC: 0.764  Perc15 and FEV_1_ %pred: 0.292  Perc15 and FEV_1_/FVC: 0.600 | %LAA-950 and FEV_1_ %pred: -0.668  %LAA-950 and FEV_1_/FVC: -0.666  %LAA-910 and FEV_1_ %pred: -0.632  %LAA-910 and FEV_1_/FVC: -0.642  MLD and FEV_1_ %pred: 0.792  MLD and FEV_1_/FVC: 0.721  Perc15 and FEV_1_ %pred: 0.352  Perc15 and FEV_1_/FVC: 0.544 |
| Bae et al, 1997 [S1] | %LAA-900 | %LAA-900 and FEV_1_ %pred: -0.52  %LAA-900 and FEV_1_/FVC: -0.33 | %LAA-900 and FEV_1_ %pred: -0.87  %LAA-900 and FEV_1_/FVC: -0.45 |
| Bafadhel et al, 2011 [S2] | Emphysema pattern, WT | No CT quantitative measurements | NA |
| Baldi et al, 2001 [S3] | %LAA-950, MLD | %LAA-950 and FEV_1_ %pred: -0.50  MLD and FEV_1_ %pred: 0.62 | NA |
| Beinert et al, 1995 [S4] | MLD | No reports on the correlation between MLD and FEV_1_ %pred or FEV_1_/FVC | NA |
| Bon et al, 2009 [18]* | %LAA-950, WA% | %LAA-950 and FEV_1_ %pred: -0.43  WA% and FEV_1_ %pred : -0.39 | NA |
| Boschetto et al, 2006 [43] | %LAA-950, MLD | %LAA-950 and FEV_1_ %pred: -0.480  MLD and FEV_1_ %pred: 0.659 | NA |
| Camiciottoli et al, 2006 [39] | %LAA-950, %LAA-910, MLD | %LAA-910 and FEV_1_/FVC: -0.57  MLD and FEV_1_/FVC: 0.60 | %LAA-910 and FEV_1_/FVC: -0.72  MLD and FEV_1_/FVC: 0.80 |
| Cavigli et al, 2009 [S5] | %LAA-950, MLD, Perc15, etc. | No reports on the correlation to FEV_1_ %pred or FEV_1_/FVC | NA |
| Cerveri et al, 2004 [S6] | %LAA-900 | NA | %LAA-900 and FEV_1_ %pred: -0.52 |
| Crausman et al, 1995 [S7] | %LAA-900 | %LAA-900 and FEV_1_ %pred: 0.34^†^  %LAA-900 and FEV_1_/FVC: 0.46^†^ | NA |
| Daghfous et al, 1993 [S8] | Visual Score | No CT quantitative measurements | NA |
| D’Anna et al, 2011 [S9] | Visual Score | No CT quantitative measurements | NA |
| Demir et al, 2005 [S10] | Visual Score | No CT quantitative measurements | NA |
| Deveci et al, 2004 [34] | WA%, T/D ratio | WA% and FEV_1_ %pred: -0.713  WA% and FEV_1_/FVC: -0.573  T/D ratio and FEV_1_ %pred: -0.735  T/D ratio and FEV_1_/FVC: -0.579 | NA |
| Dransfield et al, 2007 [19]* | %LAA-950 | %LAA-950 and FEV_1_ %pred: -0.44 (total)  %LAA-950 and FEV_1_/FVC: -0.58 (total)  %LAA-950 and FEV_1_ %pred: -0.42 (men)  %LAA-950 and FEV_1_/FVC: -0.62 (men)  %LAA-950 and FEV_1_ %pred: -0.49 (women)  %LAA-950 and FEV_1_/FVC: -0.55 (women) | NA |
| Falaschi et al, 1995 [33] | %LAA-900, MLD, Visual Score | %LAA-900 and FEV_1_ %pred: -0.80  %LAA-900 and FEV_1_/FVC: -0.86  MLD and FEV_1_ %pred: 0.76  MLD and FEV_1_/FVC: 0.81 | %LAA-900 and FEV_1_ %pred: -0.82  %LAA-900 and FEV_1_/FVC: -0.86  MLD and FEV_1_ %pred: 0.85  MLD and FEV_1_/FVC: 0.89 |
| Gelb et al, 1993 [S11] | Visual Score | No CT quantitative measurements | NA |
| Grydeland et al, 2010 [S12] | %LAA-950, -910, etc. | No reports on the correlation to FEV_1_ %pred or FEV_1_/FVC | NA |
| Grydeland et al, 2011 [S13] | %LAA-950, WT | No reports on the correlation to FEV_1_ %pred or FEV_1_/FVC | NA |
| Hasegawa et al, 2006 [20]* | WA%, Ai, WA | WA% and FEV_1_ %pred: -0.547  Ai and FEV_1_ %pred: 0.731 | NA |
| Hesselbacher et al, 2011 [29]* | %LAA-950, etc. | %LAA-950 and FEV_1_/FVC: -0.71 (current smoker)  %LAA-950 and FEV_1_/FVC: -0.78 (former smoker) | NA |
| Heussel et al, 2009 [36] | %LAA-950, MLD, Perc15, LV, etc. | %LAA-950 and FEV_1_ %pred : -0.35  %LAA-950 and FEV_1_/FVC: -0.63  MLD and FEV_1_ %pred : 0.43  MLD and FEV_1_/FVC: 0.69  Perc15 and FEV_1_ %pred : 0.34  Perc15 and FEV_1_/FVC: 0.62  LV and FEV_1_ %pred : -0.02  LV and FEV_1_/FVC: -0.58 | NA |
| Iwasawa et al, 2007 [35] | %LAA-950, MLD | %LAA-950 and FEV_1_: -0.661  %LAA-950 and FEV_1_/FVC: -0.745  MLD and FEV_1_: 0.636  MLD and FEV_1_/FVC: 0.782 | NA |
| Iwasawa et al, 2011 [31]* | %LAA-950 | %LAA-950 and FEV_1_ %pred: -0.43  %LAA-950 and FEV_1_/FVC: -0.49 | NA |
| Jin et al, 2007 [32] | %LAA-960, %LAA-950,  %LAA-910, %LAA-900, etc. | %LAA-960 and FEV_1_ %pred: -0.501  %LAA-960 and FEV_1_/FVC: -0.465  %LAA-950 and FEV_1_ %pred: -0.534  %LAA-950 and FEV_1_/FVC: -0.513  %LAA-910 and FEV_1_ %pred: -0.516  %LAA-910 and FEV_1_/FVC: -0.584  %LAA-900 and FEV_1_ %pred: -0.470  %LAA-900 and FEV_1_/FVC: -0.563 | %LAA-910 and FEV_1_ %pred: -0.562  %LAA-910 and FEV_1_/FVC: -0.506  %LAA-900 and FEV_1_ %pred: -0.571  %LAA-900 and FEV_1_/FVC: -0.523 |
| Jogi et al, 2011 [S14] | Emphysema percentage | No CT quantitative measurements | NA |
| Kim et al, 2009 [S15] | %LAA-950, WT | %LAA-950 and FEV_1_ %pred: -0.07  WT and FEV_1_ %pred: -0.12 | NA |
| Kosciuch et al, 2009 [11] | WA%, Ai, WA | No reports on the correlation with FEV_1_ %pred or FEV_1_/FVC in COPD subgroup | NA |
| Lamers et al, 1994 [8] | Visual score | No CT quantitative measurements | No CT measurements |
| Leader et al, 2008 [21]* | WA%, Ai, Ao, WA | WA% and FEV_1_ %pred: -0.584  Ai and FEV_1_ %pred: 0.540  Ao and FEV_1_ %pred: 0.410  WA and FEV_1_ %pred: 0.172 | NA |
| Leader et al, 2009 [S16] | WA%, Ai, Ao, WA, etc. | WA% and FEV_1_ %pred: -0.238  WA% and FEV_1_/FVC: -0.180  Ai and FEV_1_ %pred: 0.286  Ai and FEV_1_/FVC: 0.237  Ao and FEV_1_ %pred: 0.149  Ao and FEV_1_/FVC: 0.128  WA and FEV_1_ %pred: -0.007  WA and FEV_1_ %pred: -0.001 | NA |
| Lee et al, 2008 [22]* | %LAA-950, MLD, Ai, WA, WA% | %LAA-950 and FEV_1_ %pred: -0.547  MLD and FEV_1_ %pred: 0.439  WA% and FEV_1_ %pred: -0.044 | %LAA-950 and FEV_1_ %pred: -0.553  MLD and FEV_1_ %pred: 0.619 |
| Lee et al, 2011 [S17] | %LAA-950 | No reports on the correlation with FEV_1_ %pred or FEV_1_/FVC | No reports on the correlation with FEV_1_ %pred or FEV_1_/FVC |
| Lee et al, 2011 [S18] | %LAA-950, MLD, LV, etc. | No reports on the correlation with FEV_1_ %pred or FEV_1_/FVC | No reports on the correlation with FEV_1_ %pred or FEV_1_/FVC |
| Li et al, 2009 [S19] | LV, etc. | LV and FEV_1_ %pred: 0.315  LV and FEV_1_/FVC: 0.191 | LV and FEV_1_ %pred: -0.616  LV and FEV_1_/FVC: -0.543 |
| Madani et al, 2010 [S20] | %LAA-980 to -900(step 10%)  Perc1 to 18 (step 2-3%) | No reports on the correlation with FEV_1_ %pred or FEV_1_/FVC |  |
| Marquez-Martin et al , 2011 [S21] | Visual score | No CT quantitative measurements | NA |
| Matsuoda et al, 2007 [14] | %LAA-950, %LAA-900 | %LAA-950 and FEV_1_ %pred: -0.471  %LAA-950 and FEV_1_/FVC: -0.428  %LAA-900 and FEV_1_ %pred: -0.404  %LAA-900 and FEV_1_/FVC: -0.320 | %LAA-950 and FEV_1_ %pred: -0.602  %LAA-950 and FEV_1_/FVC: -0.554  %LAA-900 and FEV_1_ %pred: -0.618  %LAA-900 and FEV_1_/FVC: -0.525 |
| Matsuoda et al, 2008 [S22] | Ai, etc. | Ai and FEV_1_ %pred: 0.26  Ai and FEV_1_/FVC: 0.28 | Ai and FEV_1_ %pred: 0.63  Ai and FEV_1_/FVC: 0.64 |
| Matsuoda et al, 2008 [S23] | Percentage between two thresholds | No CT quantitative measurements | No CT quantitative measurements |
| Mets et al, 2011 [S24] | log%LAA-950, Perc15,etc. | log%LAA-950 and logFEV_1_: 0.53^†^  log%LAA-950 and FEV_1_/FVC: 0.61^†^  Perc15 and logFEV_1_: 0.44^†^  Perc15 and FEV_1_/FVC: 0.53^†^ | NA |
| Mishima et al, 1999 [S25] | %LAA-960 | %LAA-960 and FEV_1_ %pred: -0.320  %LAA-960 and FEV_1_/FVC: -0.528 | NA |
| Mohamed Hoesein, 2011 [45] | %LAA-950, Perc15 | %LAA-950 and FEV_1_ %pred: -0.16  %LAA-950 and FEV_1_/FVC: -0.42  Perc15 and FEV_1_ %pred: 0.12  Perc15 and FEV_1_/FVC: 0.39 | NA |
| Moron et al, 2004 [S26] | Visual score | No CT quantitative measurements | NA |
| Moroni et al, 2001 [S27] | %LAA-910, MLD | No reports on the correlation with FEV_1_ %pred or FEV_1_/FVC | %LAA-910 and FEV_1_/FVC: -0.78  MLD and FEV_1_/FVC: 0.85 |
| Nakano et al, 1999 [S28] | %LAA-960 | %LAA-960 and FEV_1_: -0.492  %LAA-960 and FEV_1_/FVC: -0.622 | NA |
| Nakano et al, 2000 [3] | %LAA-960  WA%, Ai, Ao, T/D ratio, etc. | %LAA-960 and FEV_1_ %pred: -0.529  %LAA-960 and FEV_1_/FVC: -0.650  WA% and FEV_1_ %pred: -0.338  Ai and FEV_1_ %pred: 0.273  Ao and FEV_1_ %pred: 0.195  WA% and FEV_1_/FVC -0.192 | NA |
| O'Donnel et al, 2004 [40] | %LAA-950, MLD | %LAA-950 and FEV_1_ %pred: -0.45  MLD and FEV_1_ %pred: 0.38 | %LAA-950 and FEV_1_ %pred: -0.52  MLD and FEV_1_ %pred: 0.63 |
| Ohara et al, 2006 [S29] | %LAA-960, WA%, Ai, Ao, T/D ratio, etc. | Separately reported by lung fields  %LAA-960 and FEV_1_ %pred: -0.331 (upper)  %LAA-960 and FEV_1_/FVC: -0.222 (upper)  %LAA-960 and FEV_1_ %pred: -0.487 (lower)  %LAA-960 and FEV_1_/FVC: -0.491 (lower)  WA% and FEV_1_ %pred: -0.336 (upper)  WA% and FEV_1_/FVC: -0.280 (upper)  WA% and FEV_1_ %pred: -0.339 (lower)  WA% and FEV_1_/FVC: -0.357 (lower) | NA |
| Ohno et al, 2011 [30]* | WA%, etc. | WA% and FEV_1_ %pred: -0.69  WA% and FEV_1_/FVC: -0.59 | NA |
| Orlandi et al, 2004 [15] | %LAA-950, -910, MLD | %LAA-950 and FEV_1_ %pred: -0.59  %LAA-950 and FEV_1_/FVC: -0.65  Separately reported by radiation dose  %LAA-910 and FEV_1_ %pred: -0.91 (normal)  %LAA-910 and FEV_1_/FVC: -0.81 (normal)  MLD and FEV_1_ %pred: 0.77 (normal)  MLD and FEV_1_/FVC: 0.78 (normal)  %LAA-910 and FEV_1_ %pred: -0.87 (low)  %LAA-910 and FEV_1_/FVC: -0.81 (low)  MLD and FEV_1_ %pred: 0.57 (low)  MLD and FEV_1_/FVC: -0.36 (low) | NA |
| Orlandi et al, 2005 [S30] | %LAA-950, MLD, WA%, WA, T/D ratio | %LAA-950 and FEV_1_ %pred: -0.42  %LAA-950 and FEV_1_/FVC: -0.50  MLD and FEV_1_ %pred: 0.40  MLD and FEV_1_/FVC: 0.59  WA% and FEV_1_ %pred: -0.04  WA% and FEV_1_/FVC: -0.009 | NA |
| Park et al, 2008 [23]* | %LAA-950 | %LAA-950 and FEV_1_ %pred: -0.46  %LAA-950 and FEV_1_/FVC: -0.67 | NA |
| Patel et al, 2008 [2] | %LAA-950, %LAA-910,  Visual score | %LAA-950 and FEV_1_ %pred: -0.31  %LAA-950 and FEV_1_/FVC: -0.41  No reports on the correlation between %LAA-910 and FEV_1_ %pred or FEV_1_/FVC | NA |
| Pauls et al, 2010 [24]* | %LAA-950, LV | %LAA-950 and FEV_1_ %pred: -0.360  LV and FEV_1_ %pred: -0.162 | NA |
| Pescarolo et al, 2008 [S31] | Visual score | No CT quantitative measurements | NA |
| Petersen et al, 2010 [S32] | WA%, Ai, Ao | No reports on the correlation with FEV_1_ %pred or FEV_1_/FVC in COPD patients | NA |
| Sandek et al, 2002 [S33] | %LAA-910, MLD | %LAA-910 and FEV_1_ %pred: -0.69  %LAA-910 and FEV_1_/FVC: -0.76  MLD and FEV_1_ %pred: 0.36  MLD and FEV_1_/FVC: 0.54 | %LAA-910 and FEV_1_ %pred: -0.83  %LAA-910 and FEV_1_/FVC: -0.87 |
| Scichilone et al, 2008 [S34] | MLD, etc | No reports on the correlation between MLD and FEV_1_ %pred or FEV_1_/FVC | NS |
| Shaker et al, 2005 [37] | %LAA-910, Perc15, etc. | %LAA-910 and FEV_1_ %pred: -0.62  %LAA-910 and FEV_1_/FVC: -0.62  Perc15 and FEV_1_ %pred: 0.62  Perc15 and FEV_1_/FVC: 0.61 | NA |
| Sorensen et al, 2010 [S35] | Emphysema pattern | No CT quantitative measurements | NA |
| Spiropoulos et al, 2003 [S36] | %LAA-910 | No reports on the correlation with FEV_1_ %pred or FEV_1_/FVC | No reports on the correlation to FEV_1_ %pred or FEV_1_/FVC |
| Torres et al, 2011 [S37] | %LAA-960 | %LAA-960 and FEV_1_ %pred: 0.14  %LAA-960 and FEV_1_/FVC: -0.24 | NA |
| Tsushima et al, 2010 [S38] | %LAA-960, Visual score | %LAA-960 and FEV_1_/FVC: -0.29 | NA |
| Van Der Lee et al, 2006 [S39] | %LAA-950, LV | %LAA-950 and FEV_1_ %pred: 0.3^†^  No reports on the correlation between LV and FEV_1_ %pred or FEV_1_/FVC | NA |
| Washko et al, 2008 [12] | %LAA-950, -910, MLD, Perc15, etc | %LAA-950 and FEV_1_ %pred: -0.09  %LAA-950 and FEV_1_/FVC: -0.09  %LAA-910 and FEV_1_ %pred: -0.20  %LAA-910 and FEV_1_/FVC: -0.19  MLD and FEV_1_ %pred: 0.18  MLD and FEV_1_/FVC: 0.21  Perc15 and FEV_1_ %pred: 0.09  Perc15 and FEV_1_/FVC: 0.12 | NA |
| Washko et al, 2009 [25]* | %LAA-950, WA%, Ai, WT | WA% and FEV_1_ %pred: -0.28  WA% and FEV_1_/FVC: -0.014  Ai and FEV_1_ %pred: 0.14  Ai and FEV_1_FVC: 0.07  WT and FEV_1_ %pred: -0.13  WT and FEV_1_/FVC: -0.05 | NA |
| Watanuki et al, 1994 [S40] | MLD | MLD and FEV_1_ %pred: 0.72 | NA |
| Yamashiro et al, 2010 [26]* | %LAA-950, MLD, LV, etc. | %LAA-950 and FEV_1_ %pred: -0.625  %LAA-950 and FEV_1_/FVC: -0.713  MLD and FEV_1_.%pred: 0.494  MLD and FEV_1_/FVC: 0.562  LV and FEV_1_ %pred: -0.010  LV and FEV_1_/FVC: -0.198 | %LAA-950 and FEV_1_ %pred: -0.637  %LAA-950 and FEV_1_/FVC: -0.729  MLD and FEV_1_.%pred: 0.661  MLD and FEV_1_/FVC: 0.743  LV and FEV_1_ %pred: -0.406  LV and FEV_1_/FVC: -0.588 |
| Yamashiro et al, 2010 [28]* | %LAA-950, WA%, Ai, etc. | %LAA-950 and FEV_1_ %pred: -0.460  WA% and FEV_1_ %pred: -0.470  Ai and FEV_1_ %pred: 0.450 | NA |
| Yamashiro et al, 2011 [S41] | Kurtosis and skewness | No CT quantitative measurements | No CT quantitative measurements |
| Zampatori et al, 1997 [S42] | %LAA-900, Visual score | No reports on the correlation with FEV_1_ %pred or FEV_1_/FVC | %LAA-900 and FEV_1_ %pred: -0.65 |
| Zampatori et al, 2001 [S43] | Visual score | No CT measurements | NA |
| Zampatori et al, 2001 [S44] | %LAA-900, LV, Visual score | LV and FEV_1_ %pred: -0.49  LV and FEV_1_/FVC: -0.69 | NA |
| Zampatori et al, 2002 [S45] | %LAA-900, MLD, LV,  Visual score | %LAA-900 and FEV_1_ %pred: -0.53 (Scan 1)  %LAA-900 and FEV_1_ %pred: -0.56(Scan 2)  %LAA-900 and FEV_1_/FVC: -0.79 (Scan 1)  %LAA-900 and FEV_1_/FVC: -0.80 (Scan 2)  No reports on the correlation between MLD, LV and FEV_1_ %pred or FEV_1_/FVC | NA |
| Zaporozhan et al, 2005 [41] | %-LAA-950, MLD, LV, etc | No reports on the correlation with FEV_1_ %pred or FEV_1_/FVC | No reports on the correlation to FEV_1_ %pred or FEV_1_/FVC |
| Zhang et al, 2008 [27]* | %-LAA-950, -910, MLD | %LAA-950 and FEV_1_ %pred: -0.520  %LAA-950 and FEV_1_/FVC: -0.626  MLD and FEV_1_ %pred: 0.416  MLD and FEV_1_/FVC: 0.512  %LAA-910 and FEV_1_ %pred: -0.437  %LAA-910 and FEV_1_/FVC: -0.548 | NA |

NA = Not available; %LAA = Percentage low attenuation area; MLD = Mean lung density; LV = Lung volume; Perc = Percentile point of lung density; WA% = Airway wall area percentage; Ai = Airway lumen area; Ao = Total airway area; WT = Wall thickness; T/D radio = Ratio of airway wall thickness to total diameter.

* Included in the meta-analysis.

† Expressed as *r^2^*.
