# Supplementary material for: Morphological measurements in computed tomography correlate with airflow obstruction in chronic obstructive pulmonary disease: systematic review and meta-analysis
Source: Eur Radiol. 2012 Jun 15;22(10):2085–93. doi: 10.1007/s00330-012-2480-8 (PMC3431473; doi:10.1007/s00330-012-2480-8)
Supplement: Supplementary file 4 — (DOC 179 kb) [file 330_2012_2480_MOESM4_ESM.doc]

**Electronic supplementary table 4 Assessment of Study Quality, by the Quality Assessment of Diagnostic Accuracy Studies (QUADAS) tool**

| **Study, Year (Reference)** | **Item1: Representative patient sample** | **Item2: Selection criteria clearly described** | **Item3: Acceptable reference standard*** | **Item4: Acceptable delay between tests^†^** | **Item5: Partial verification avoided** | **Item6: Differential verification avoided** | **Item7: Incorporation avoided** | **Item8: Adequate index test description*** | **Item9:Adequate reference standard description*** | **Item10: Index test blinded to reference standard*** | **Item11: Reference standard blinded to index test*** | **Item12: Clinical data available as in practice** | **Item13: Uninterpretable test results reported** | **Item14: Withdrawals explained** | **Score^†^** |
| --- | --- | --- | --- | --- | --- | --- | --- | --- | --- | --- | --- | --- | --- | --- | --- |
| Akira et al, 2009 [13] | Y | Y | Y | Y | Y | Y | Y | Y | Y | U | U | Y | Y | Y | 13.0 |
| Bon et al, 2009 [18] | Y | Y | Y | U | Y | Y | Y | Y | Y | U | Y | Y | Y | Y | 13.0 |
| Dransfield et al, 2007 [19] | Y | Y | Y | U | Y | Y | Y | Y | Y | U | U | Y | Y | Y | 12.5 |
| Hasegawa et al, 2006 [20] | Y | Y | Y | Y | Y | Y | Y | Y | Y | Y | U | Y | Y | Y | 13.5 |
| Hesselbacher et al, 2011 [29] | Y | Y | Y | U | Y | Y | Y | Y | Y | U | Y | Y | Y | Y | 13.0 |
| Iwasawa et al, 2011 [31] | Y | Y | Y | Y | Y | Y | Y | Y | Y | Y | U | Y | Y | Y | 13.5 |
| Leader et al, 2008 [21] | Y | Y | Y | U | Y | Y | Y | Y | Y | U | U | Y | Y | Y | 12.5 |
| Lee et al, 2008 [22] | Y | Y | Y | Y | Y | Y | Y | Y | Y | Y | U | Y | Y | Y | 13.5 |
| Ohno et al, 2011 [30] | Y | Y | Y | Y | Y | Y | Y | Y | Y | U | U | Y | Y | Y | 13.0 |
| Park et al, 2008 [23] | Y | Y | Y | Y | Y | Y | Y | Y | Y | U | U | Y | Y | Y | 13.0 |
| Pauls et al, 2010 [24] | Y | Y | Y | Y | Y | Y | Y | Y | Y | Y | U | Y | Y | Y | 13.5 |
| Washko et al, 2009 [25] | Y | Y | Y | U | Y | Y | Y | Y | Y | Y | U | Y | Y | Y | 13.0 |
| Yamashiro et al, 2010 [26] | Y | Y | Y | U | Y | Y | Y | Y | Y | U | U | Y | Y | Y | 12.5 |
| Yamashiro et al, 2010 [28] | Y | Y | Y | U | Y | Y | Y | Y | Y | Y | U | Y | Y | Y | 13.0 |
| Zhang et al, 2008 [27] | Y | Y | Y | Y | Y | Y | Y | Y | Y | U | U | Y | Y | Y | 13.0 |
| Item number (Yes) | 15 | 15 | 15 | 8 | 15 | 15 | 15 | 15 | 15 | 6 | 2 | 15 | 15 | 15 |  |
| Item number (No) | 0 | 0 | 0 | 0 | 0 | 0 | 0 | 0 | 0 | 0 | 0 | 0 | 0 | 0 |  |
| Item number (Unknown) | 0 | 0 | 0 | 7 | 0 | 0 | 0 | 0 | 0 | 9 | 13 | 0 | 0 | 0 |  |

Y = Yes; U = Unclear; N = No.

* CT qualification was considered as index test in item 8, 10, 11; PFT was considered as reference standard in item 3, 9, 10, 11.

† Maximum delay of 30 days between CT and PFT was considered as acceptable.

‡ For each article, a quality score was accumulated by assigning 1 point to “yes” item, 0.5 point to “unclear” item, and 0 point for “no” item.
