# Supplementary material for: Morphological measurements in computed tomography correlate with airflow obstruction in chronic obstructive pulmonary disease: systematic review and meta-analysis
Source: Eur Radiol. 2012 Jun 15;22(10):2085–93. doi: 10.1007/s00330-012-2480-8 (PMC3431473; doi:10.1007/s00330-012-2480-8)
Supplement: Supplementary file 5 — (DOC 100 kb) [file 330_2012_2480_MOESM5_ESM.doc]

**Electronic supplementary table 5 Bias analysis in the meta-analysis**

|  | **Heterogeneity** | | | **Publication bias** | | | **Meta-regression for gender*** |
| --- | --- | --- | --- | --- | --- | --- | --- |
|  | **Q value** | ***P*(Q)** | **I^2^ ,%** | **Fail-safe N** | **Begg and Mazumdar rank correlation, *P*** | **Egger’s regression, *P*** | ***P*(Slope)** |
| **Inspiration** |  |  |  |  |  |  |  |
| %LAA-950 and FEV_1_ %pred | 11.65 | 0.11 | 39.9 | 451 | 0.62 | 0.22 | 0.16 |
| %LAA-950 and FEV_1_/FVC | 10.22 | 0.12 | 41.3 | 614 | 0.45 | 0.31 | 0.71 |
| MLD and FEV_1_ %pred | 6.08 | 0.11 | 50.6 | 97 | 0.50 | 0.14 | 0.22 |
| MLD and FEV_1_/FVC | 8.29 | 0.02 | 75.9 | 172 | 0.60 | 0.77 | 0.05 |
| WA% and FEV_1_ %pred | 8.50 | 0.04 | 64.7 | 255 | 0.73 | 0.60 | IS |
| **Expiration** |  |  |  |  |  |  |  |
| %LAA-950 and FEV_1_ %pred | 0.81 | 0.67 | 0 | 84 | 0.12 | 0.21 | 0.85 |
| %LAA-950 and FEV_1_/FVC | 0.38 | 0.54 | 0 | IS | IS | IS | IS |
| MLD and FEV_1_ %pred | 3.55 | 0.17 | 43.6 | 122 | 0.12 | 0.15 | 0.56 |
| MLD and FEV_1_/FVC | 0.05 | 0.82 | 0 | IS | IS | IS | IS |

FEV_1_ %pred = Predicted forced expiratory volume in the first second; FEV_1_/FVC = FEV_1_ divided by forced vital capacity; %LAA-950 = Percentage lower attenuation area than -950HU; MLD = Mean lung density; Perc15 = 15 percentile point of lung density; WA% = Wall area percentage; IS = Insufficient study numbers to perform analysis.

*: Percentage of male was considered as moderator variable.
