# Supplementary material for: Morphological measurements in computed tomography correlate with airflow obstruction in chronic obstructive pulmonary disease: systematic review and meta-analysis
Source: Eur Radiol. 2012 Jun 15;22(10):2085–93. doi: 10.1007/s00330-012-2480-8 (PMC3431473; doi:10.1007/s00330-012-2480-8)
Supplement: Supplementary file 6 — (DOC 77.5 kb) [file 330_2012_2480_MOESM6_ESM.doc]

**Electronic supplementary Fig. 4 Counting of CT measurements in the systematic review**

|  |
| --- |

%LAA = Percentage low attenuation area; MLD = Mean lung density; WA = Airway wall area; Ai = Airway lumen area; LV = Lung volume; WT = Wall thickness; Perc = Percentile point of lung density; Ao = Total airway area; T/D radio = Radio of airway wall thickness to total diameter; EV = Emphysema Volume; HU = Hounsfield unit; BSA = Body surface area; Vex = Expiration volume; Vin = Inspiration volume; EA = Expiration airway lumen area; IA = Inspiration airway lumen area; SRWA = Square root of wall area; FWHM = Full width at half maximum; SD = Standard deviation.
