# Supplementary material for: Morphological measurements in computed tomography correlate with airflow obstruction in chronic obstructive pulmonary disease: systematic review and meta-analysis
Source: Eur Radiol. 2012 Jun 15;22(10):2085–93. doi: 10.1007/s00330-012-2480-8 (PMC3431473; doi:10.1007/s00330-012-2480-8)
Supplement: Supplementary file 7 — (DOC 76.5 kb) [file 330_2012_2480_MOESM7_ESM.doc]

**Electronic supplementary Fig. 5 Counting of pulmonary function test parameters in the systematic review**

|  |
| --- |

%pred = Predicted percentage; FEV_1_ = Forced expiratory volume in the first second; FEF = Forced expiratory flow; VA = Alveolar volume; TLC = Total lung capacity; RV = Residual volume; DLCO = D[iffusing capacity](http://en.wikipedia.org/wiki/Diffusing_capacity) of the [lung](http://en.wikipedia.org/wiki/Lung) for [carbon monoxide](http://en.wikipedia.org/wiki/Carbon_monoxide); VC = [Vital capacity](http://en.wikipedia.org/wiki/Vital_capacity); TGV = Thoracic gas volume; FVC = Forced vital capacity; PEFR = Peak expiratory flow rate; KCO = Carbon monoxide transfer coefficient; MEF = Maximal expiratory flow; IC = Inspiratory capacity; FRC = [Functional residual capacity](http://en.wikipedia.org/wiki/Functional_Residual_Capacity); FIF = Forced inspiratory flow.
