# Supplementary figures and images for: Morphological measurements in computed tomography correlate with airflow obstruction in chronic obstructive pulmonary disease: systematic review and meta-analysis
Source: Eur Radiol. 2012 Jun 15;22(10):2085–93. doi: 10.1007/s00330-012-2480-8 (PMC3431473; doi:10.1007/s00330-012-2480-8)

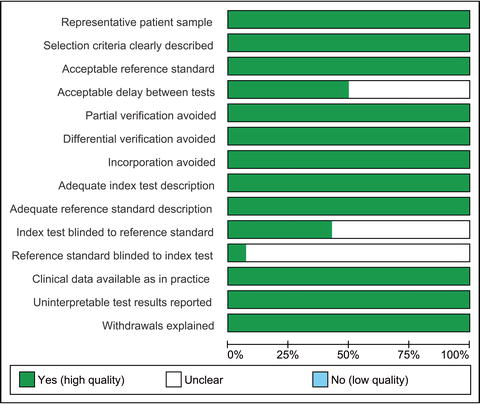

Supplement: Supplementary file 8 — (JPEG 36 kb) [file 330_2012_2480_MOESM8_ESM.jpg]
