# Supplementary material for: Morphological measurements in computed tomography correlate with airflow obstruction in chronic obstructive pulmonary disease: systematic review and meta-analysis
Source: Eur Radiol. 2012 Jun 15;22(10):2085–93. doi: 10.1007/s00330-012-2480-8 (PMC3431473; doi:10.1007/s00330-012-2480-8)
Supplement: Supplementary file 9 — (DOC 36.5 kb) [file 330_2012_2480_MOESM9_ESM.doc]

**Electronic supplementary References**

1. Bae KT, Slone RM, Gierada DS, Yusen RD, Cooper JD (1997) Patients with emphysema: quantitative CT analysis before and after lung volume reduction surgery. Radiology 203:705–714
2. Bafadhel M, Umar I, Gupta S et al (2011) The role of computed tomography in multi-dimensional phenotyping of chronic obstructive pulmonary disease. Chest 140:634–642
3. Baldi S, Miniati M, Bellina CR et al (2001) Relationship between extent of pulmonary emphysema by high-resolution computed tomography and lung elastic recoil in patients with chronic obstructive pulmonary disease. Am J Respir Crit Care Med 164:585–589
4. Beinert T, Behr J, Mehnert F et al (1995) Spirometrically controlled quantitative CT for assessing diffuse parenchymal lung disease. J Comput Assist Tomogr 19:924–931
5. Cavigli E, Camiciottoli G, Diciotti S et al (2009) Whole-lung densitometry versus visual assessment of emphysema. Eur Radiol 19:1686–1692
6. Cerveri I, Dore R, Corsico A et al (2004) Assessment of emphysema in COPD—a functional and radiologic study. Chest 125:1714–1718
7. Crausman RS, Ferguson G, Irvin CG, Make B, Newell JD (1995) Quantitative chest computed tomography as a means of predicting exercise performance in severe emphysema. Acad Radiol 2:463–469
8. Daghfous J, Beji M, Kanoun N et al (1993) Pulmonary emphysema quantification using computed tomography and correlations with respiratory function tests. Rev Mal Respir 10:299–305
9. D'Anna SE, Asnaghi R, Caramori G et al (2011) High-resolution computed tomography quantitation of emphysema is correlated with selected lung function values in stable COPD. Respiration. doi:10.1159/000329871
10. Demir T, Ikitimur H, Akpinar Tekgunduz S et al (2005) The relationship between pulmonary function tests, thorax HRCT, and quantitative ventilation-perfusion scintigraphy in chronic obstructive pulmonary disease. Tüberkülozve Toraks Dergisi 53:347–353
11. Gelb AF, Schein M, Kuei J et al (1993) Limited contribution of emphysema in advanced chronic obstructive pulmonary disease. Am Rev Respir Dis 147:1157–1161
12. Grydeland TB, Dirksen A, Coxson HO et al (2010) Quantitative computed tomography measures of emphysema and airway wall thickness are related to respiratory symptoms. Am J Respir Crit Care Med 181:353–359
13. Grydeland TB, Thorsen E, Dirksen A et al (2011) Quantitative CT measures of emphysema and airway wall thickness are related to D(L)CO. Respir Med 105:343–351
14. Jogi J, Ekberg M, Jonson B, Bozovic G, Bajc M (2011) Ventilation/perfusion SPECT in chronic obstructive pulmonary disease: an evaluation by reference to symptoms, spirometric lung function and emphysema, as assessed with HRCT. Eur J Nucl Med Mol Imaging 38:1344–1352
15. Kim WJ, Silverman EK, Hoffman E et al (2009) CT metrics of airway disease and emphysema in severe COPD. Chest 136:396–404
16. Leader JK, Bin Z, Fuhrman CR et al (2009) Association between lung function and airway wall density. Proc SPIE Int Soc Opt Eng 72622J:72629
17. Lee JS, Ra SW, Chae EJ et al (2011) Validation of the lower limit of normal diffusing capacity for detecting emphysema. Respiration 81:287–293
18. Lee HJ, Seo JB, Chae EJ et al (2011) Tracheal morphology and collapse in COPD: Correlation with CT indices and pulmonary function test. Eur J Radiol 80:e531–e535
19. Li S, Chen L, Du JM, Hua SC (2009) Application of measurement of lung volumes from MSCT images in evaluation of pulmonary function of patients with chronic obstructive pulmonary disease. J Jilin Univ Med Ed (Med Ed) 35:1167–1171
20. Madani A, Van Muylem A, Gevenois PA (2010) Pulmonary emphysema: effect of lung volume on objective quantification at thin-section CT. Radiology 257:260–268
21. Marquez-Martin E, Ramos PC, Lopez-Campos JL et al (2011) Components of physical capacity in patients with chronic obstructive pulmonary disease: relationship with phenotypic expression. Int J Chron Obstruct Pulmon Dis 6:105–112
22. Matsuoka S, Kurihara Y, Yagihashi K, Hoshino M, Nakajima Y (2008) Airway dimensions at inspiratory and expiratory multisection CT in chronic obstructive pulmonary disease: Correlation with airflow limitation. Radiology 248:1042–1049
23. Matsuoka S, Kurihara Y, Yagihashi K, Hoshino M, Watanabe N, Nakajima Y (2008) Quantitative assessment of air trapping in chronic obstructive pulmonary disease using inspiratory and expiratory volumetric MDCT. Am J Roentgenol 190:762–769
24. Mets OM, Murphy K, Zanen P et al (2011) The relationship between lung function impairment and quantitative computed tomography in chronic obstructive pulmonary disease. Eur Radiol. doi:10.1007/s00330-00011-02237-00339
25. Mishima M, Itoh H, Sakai H et al (1999) Optimized scanning conditions of high resolution CT in the follow-up of pulmonary emphysema. J Comput Assist Tomogr 23:380–384
26. Moron K, Liebhart J, Liebhart E, Ciesielska A, Badowski R (2004) Structural and functional lung changes in patients with COPD and asthma—the benefit of quantitative HRCT assessment. Adv Clin Exp Med 13:59–66
27. Moroni C, Mascalchi M, Bartolucci M, Camiciottoli G, Pistolesi M, Villari N (2001) High resolution and spirometric synchronization computerized tomography in chronic obstructive bronchopneumopathy. Radiol Med 101:25–30
28. Nakano Y, Sakai H, Muro S et al (1999) Comparison of low attenuation areas on computed tomographic scans between inner and outer segments of the lung in patients with chronic obstructive pulmonary disease: incidence and contribution to lung function. Thorax 54:384–389
29. Ohara T, Hirai T, Sato S et al (2006) Comparison of airway dimensions in different anatomic locations on chest CT in patients with COPD. Respirology 11:579–585
30. Orlandi I, Moroni C, Camiciottoli G et al (2005) Chronic obstructive pulmonary disease: Thin-section CT measurement of airway wall thickness and lung attenuation. Radiology 234:604–610
31. Pescarolo M, Sverzellati N, Verduri A et al (2008) How much do GOLD stages reflect CT abnormalities in COPD patients? Radiol Med 113:817–829
32. Petersen J, Pechin L, Nielsen M et al (2010) Quantitative analysis of airway abnormalities in CT. Proc SPIE Int Soc Opt Eng 76241 S:76212
33. Sandek K, Bratel T, Lagerstrand L, Rosell H (2002) Relationship between lung function, ventilation-perfusion inequality and extent of emphysema as assessed by high-resolution computed tomography. Respir Med 96:934–943
34. Scichilone N, La Sala A, Bellia M et al (2008) The airway response to deep inspirations decreases with COPD severity and is associated with airway distensibility assessed by computed tomography. J Appl Physiol 105:832–838
35. Sorensen L, Shaker SB, de Bruijne M (2010) Quantitative analysis of pulmonary emphysema using local binary patterns. IEEE Trans Med Imaging 29:559–569
36. Spiropoulos K, Trakada G, Kalamboka D et al (2003) Can high resolution computed tomography predict lung function in patients with chronic obstructive pulmonary disease? Lung 181:169–181
37. de Torres JP, Bastarrika G, Zagaceta J et al (2011) Emphysema presence, severity, and distribution has little impact on the clinical presentation of a cohort of patients with mild to moderate COPD. Chest 139:36–42
38. Tsushima K, Sone S, Fujimoto K et al (2010) Identification of occult parechymal disease such as emphysema or airway disease using screening computed tomography. COPD 7:117–125
39. van der Lee I, van Es HW, Noordmans HJ, van den Bosch JM, Zanen P (2006) Alveolar volume determined by single-breath helium dilution correlates with the high-resolution computed tomography-derived nonemphysematous lung volume. Respiration 73:468–473
40. Watanuki Y, Suzuki S, Nishikawa M, Miyashita A, Okubo T (1994) Correlation of quantitative CT with selective alveolobronchogram and pulmonary function tests in emphysema. Chest 106:806–813
41. Yamashiro T, Matsuoka S, Estepar RSJ et al (2011) Kurtosis and skewness of density histograms on inspiratory and expiratory CT scans in smokers. COPD 8:13–20
42. Zompatori M, Battaglia M, Rimondi MR et al (1997) Quantitative assessment of pulmonary emphysema with computerized tomography. Comparison of the visual score and high resolution computerized tomography, expiratory density mask with spiral computerized tomography and respiratory function tests. Radiol Med 93:374–381
43. Zompatori M, Fasano L, Battista G, Pacilli AM (2001) Longitudinal follow-up evaluation of pulmonary emphysema using high-resolution Computed Tomography (HRCT) study and pulmonary function tests. Radiol Med 102:122–126
44. Zompatori M, Fasano L, Pacilli AM, Battista G, Cavina M, Pezzi S (2001) Automatic evaluation of total lung capacity and of emphysema involvement with spiral computerized tomography (CT) in obstructive pneumonia. Radiol Med 101:18–24
45. Zompatori M, Fasano L, Mazzoli M et al (2002) Spiral CT evaluation of pulmonary emphysema using a low-dose technique. Radiol Med 104:13–24
